# Birth weight and grip strength in young Swedish males: a longitudinal matched sibling analysis and across all body mass index ranges

**DOI:** 10.1038/s41598-019-46200-0

**Published:** 2019-07-04

**Authors:** Viktor H. Ahlqvist, Margareta Persson, Francisco B. Ortega, Per Tynelius, Cecilia Magnusson, Daniel Berglind

**Affiliations:** 1 Department of Public Health Sciences, Karolinska Institutet, Solna, Sweden; 2 Umeå University, Department of Nursing, Umeå, Sweden; 3 PROFITH “PROmoting FITness and Health through physical activity” research group, Department of Physical Education and Sports, Faculty of Sport Sciences, University of Granada, Granada, Spain; 4 Department of Biosciences and Nutrition, Karolinska Institutet, Solna, Sweden; 5 Centre for Epidemiology and Community Medicine, Stockholm County Council, Stockholm, Sweden

**Keywords:** Paediatric research, Epidemiology

## Abstract

Low birth weight is associated with a lower grip strength later in life. However, associations between birth weight among infants born at-term and factors driving associations between birth weight and grip strength are largely unknown. A cohort of 144,369 young men born at-term, including 10,791 individuals who had at least one male sibling/s, were followed until conscription where they performed a grip strength test. We used linear and non-linear regression analyses in the full cohort, and fixed-effects regression analyses in the sibling cohort, to address confounding by factors that are shared between siblings. After adjustment, each unit increase in birth weight z-score was associated with increases of 17.7 (95% CI, 17.2–18.2) and 13.4 (10.1–16.6) newton grip strength, which converts to approximately 1.8 and 1.4 kilogram-force in the full and within-families cohorts, respectively. The associations did not vary with young adulthood BMI. Birth weight, within the at-term range, is robustly positively associated with grip strength in young adulthood among men across all BMI categories and associations appears to be mainly driven by factors that are not shared between siblings. These findings underline the importance of recognizing the influence of low birth weight, also within the at-term-range, on young adulthood muscle strength.

## Introduction

Low birth weight^1^, preterm and early term births^2^ are associated with an increased all-cause mortality risk, implicating that a compromised fetal environment may have long-lasting health consequences. Birth weight is not only determined by the prenatal environment, but also by genetic influences. Hence, associations between birth weight and later health outcomes may be a consequence of a compromised environment in utero or genetic factors that affect both birth weight and later health outcomes. Therefore, family studies are needed to provide further evidence of the extent to which the associations between birth weight and later health outcomes are explained by genetical factors and other family shared environmental factors. Gestational age is, in addition to birth weight, an important determinant of adult health^3,4^. It is becoming increasingly evident that infants born at-term (i.e. week 37–41) form a heterogeneous group^5^. Indeed, birth weight within the at-term range is associated with later health outcomes^6,7^. By using births within the at-term range, confounding by gestational age and/or prematurity can be ruled out. In all, these findings warrant further explorations of long‐term health markers associated to birth weight among infants born at-term.

One such important and modifiable health marker is grip strength which is associated with a wide range of health outcomes later in life, including all-cause mortality rates equivalent to that of well-established risk factors such as elevated blood pressure or body mass index (BMI)^8–10^. Furthermore, higher grip strength has been demonstrated to attenuate the increased mortality associated with an elevated BMI^11^, suggesting that grip strength has important health benefits across all BMI categories. There is a strong correlation (i.e. >0.8) between grip strength and total muscle strength in young males^12^, which makes grip strength an appropriate general indicator for overall muscle strength^13^. Muscle strength is the capacity to carry out work against a resistance, depending on several factors (e.g. muscle mass, neuromuscular function etc.). Studies have suggested that the major factor influencing the observed inverse association between grip strength and mortality is not attributable to the muscle mass per se, but to muscle strength as a marker of muscle quality^14^. Overall, current evidence from epidemiological observational studies indicate that low birth weight, across all gestational ages, is associated with lower grip strength, even after adjusting for potential confounding factors^15^. However, most previous studies on the topic have been confined to small sample sizes, grip strength comparisons between preterm birth and term‐born controls being used as a homogenous comparator and/or unable to control whether such associations are explained by shared and/or non-shared familial factors^15^.

Therefore, the aim of this study was to investigate the association between birth weight, within the at-term range, and grip strength in young males. We further investigate whether any such association is explained by factors shared between siblings, and if associations differ with BMI in young adulthood.

## Methods

### Study design

This prospective cohort study used unique personal identification numbers, assigned to each Swedish resident at birth, for record linkage of four different Swedish population-based registers: (i) the Medical Birth Register (MBR) containing validated data on >99% of all births in Sweden^16^, (ii) the Swedish Military Service Conscription Registry, (iii) the Population and Housing Censuses from 1970 and 1990 and (iv) the Multigenerational Register. The study was approved by the Regional Ethical Review Board, Stockholm (Dnr: 2016/1445-31/1).

### Study population

**Figure 1:**
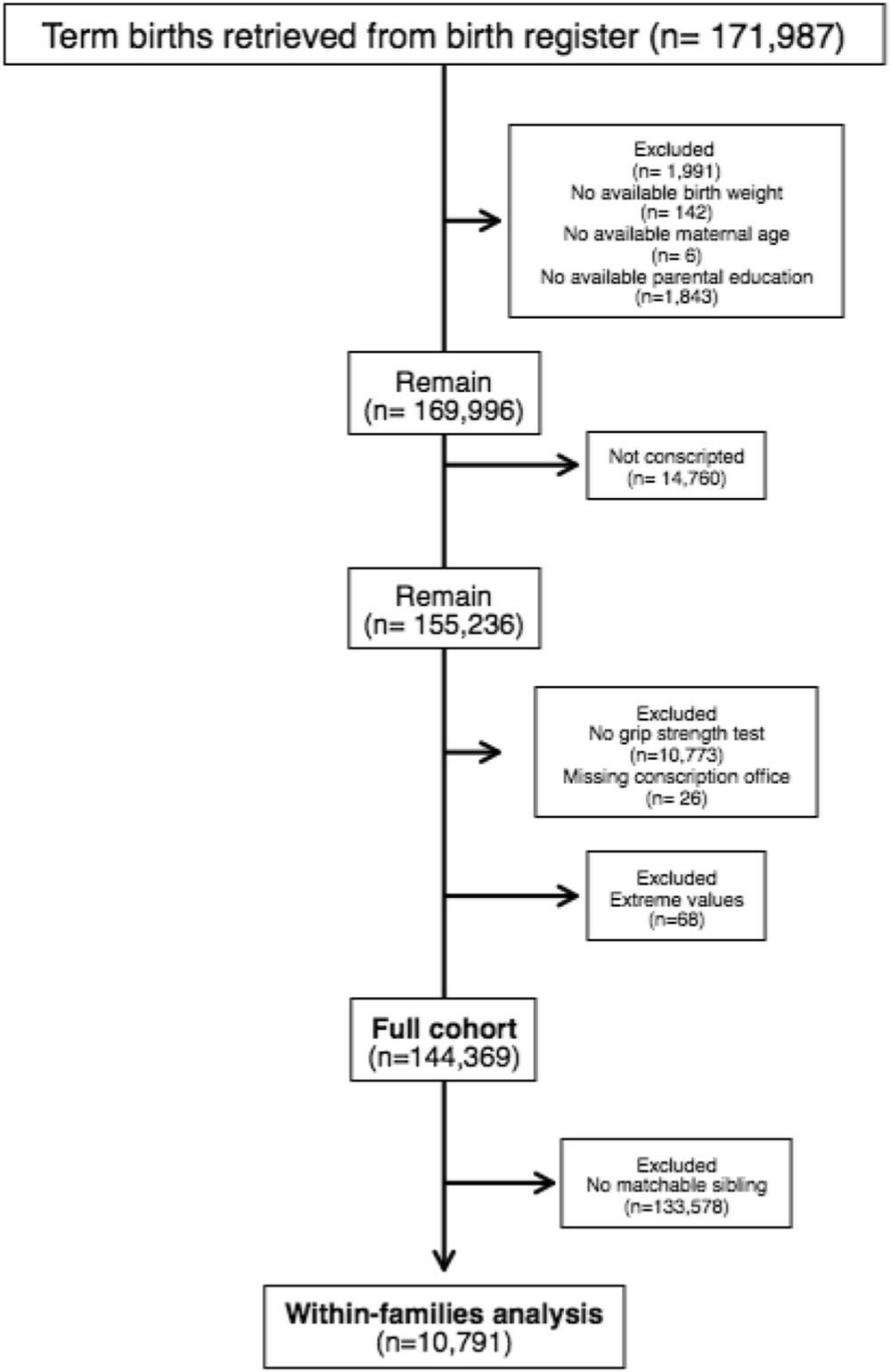
Flowchart of the derivation of the analytical sample.

All men born at-term in Sweden 1973–1976 (n = 171,987) and who conscripted for military service in 1991–1994 (n = 155,236) were eligible for inclusion in the study. In that time period, conscription was mandatory by law, and young adults were relieved from conscription only due to confinement or if they suffered from severe medical condition. The study period was chosen to match the availability of perinatal exposure data from the MBR (available from 1973) and outcome grip strength data from the conscription registry. Exclusion criteria included incomplete data (n = 10,867) including those who did not perform the grip test at conscription (n = 10,773), or extreme values (n = 68) for weight (≤40 or ≥150 kg), height (≤150 or ≥210 cm) and BMI (≤15 or ≥60 kg/m^2^) at conscription, as previously described^17^. Derivation of the analytical sample is presented in a flowchart in Fig. 1. In total, 144,369 (93%) young adults performed the grip test and were therefore included in the final analytical sample. The within-families cohort included 10,791 individuals who had one or more matchable male sibling/s.

### Exposures

All perinatal variables were derived from the MBR^16^. At-term births were defined as births between 37–41 weeks^6^ and the date of the last menstrual period was used to estimate gestational age^18^. Birth weight was measured in grams, and extreme values <300 g and >7,000 g were excluded. Gestational age specific birth weight z-scores were estimated using the total study population as the reference. For a baby born at 40 weeks, one SD corresponds to approximately 455 grams.

### Outcome – grip strength

Grip strength was measured in Newton’s (N), using standard well-validated and daily calibrated isometric dynamometer test^19^, where 9.8 N equals one kilogram force. Each dynamometer test was performed, using the dominant hand, three times and the maximum value recorded for analysis. However, if the last value in the last trial was higher than the previous trials, testing was repeated until strength values stopped increasing.

### Confounders and covariates

Standardized scales and stadiometers were used to measure weight and height at conscription, respectively^20^. BMI (kg/m^2^) was classified according to World Health Organization (WHO) categories: underweight (BMI <18.5), normal weight (BMI 18.5–24.9), overweight (BMI 25–29.9) and obese (BMI ≥30)^21^. Parental education, where the highest education achieved by any of the parents was used as a measure of household socioeconomic position, was derived from the Population and Housing Censuses. Information regarding conscription center (six centers) and age at conscription were retrieved from the Conscription Registry. The MBR was used for information on further potential confounders, such as maternal diabetes, maternal hypertension, birth by caesarean section, maternal parity and maternal age at birth.

### Statistical analyses

First, we descriptively present our cohort by dichotomizing the exposure and outcome by their respective median into four groups: (i) high grip strength/high birth weight z-score, (ii) high grip strength/low birth weight z-score, (iii) low grip strength/high birth weight z-score and (iv) low grip strength/low birth weight z-score (Table 1 and Supplementary Table 1). Second, we used linear regression models to analyze the associations between z-score birth weight, within the at-term range, and grip strength. Third, to control for shared factors (fixed effects) by full brothers within families (approximately 50% shared genetic factors) we used fixed-effects regression models (Table 2). Fourth, we stratified our crude and fully adjusted models on previously described BMI categories at conscription (Table 3). Additionally, we examined if there was any statistical interaction between continuous BMI and z-score birth weight in the association with grip-strength (data not shown).

**Table 1:** Characteristics of the Full Cohort by Birth Weight (Z-score) and Grip Strength (Newton), Swedish Males Born At-term Between 1973–1976.

Characteristic	Full cohort	High grip strength and High birth weight^a^	High grip strength and Low birth weight^a^	Low grip strength and High birth weight^a^	Low grip strength and Low birth weight^a^
	N = 144,369	N = 41,535	N = 32,723	N = 30,801	N = 39,310
*Gestational age (weeks), mean (SD)*	39.6 (1.1)	39.6 (1.1)	39.6 (1.1)	39.6 (1.1)	39.6 (1.1)
*Birth weight (g), mean (SD)*	3,573.9 (486.4)	3,953.0 (347.5)	3,245.3 (300.1)	3,901.4 (329.0)	3,190.2 (329.6)
*Handgrip strength (N), mean (SD)*	614.3 (97.4)	693.6 (70.2)	681.2 (63.4)	540.6 (51.1)	532.7 (54.3)
*BMI (kg/m²) at conscription, mean (SD)*	22.1 (3.0)	22.9 (3.0)	22.6 (3.0)	21.6 (2.9)	21.3 (2.9)
*Height (cm) at conscription, mean (SD)*	179.7 (6.5)	182.5 (6.1)	179.7 (6.0)	179.6 (6.1)	176.7 (6.1)
*Age at conscription, mean (SD)*	18.3 (0.4)	18.3 (0.4)	18.3 (0.4)	18.3 (0.4)	18.3 (0.4)
*Maternal age at birth, mean (SD)*	26.8 (4.8)	27.1 (4.8)	26.3 (4.7)	27.4 (4.9)	26.5 (4.8)
*Maternal parity, median (IQR)*	2.0 (1.0, 2.0)	2.0 (1.0, 2.0)	1.0 (1.0, 2.0)	2.0 (1.0, 2.0)	2.0 (1.0, 2.0)
*Maternal diabetes mellitus at pregnancy, n (%)*	382 (0.3%)	134 (0.3%)	46 (0.1%)	110 (0.4%)	92 (0.2%)
*Birth by cesarean section, n (%)*	9,556 (6.6%)	2,384 (5.7%)	2,119 (6.5%)	1,929 (6.3%)	3,124 (7.9%)
*Maternal hypertension at pregnancy, n (%)*	60 (<1%)	16 (<1%)	15 (<1%)	12 (<1%)	17 (<1%)
***Parental highest level of education, n (%)***					
Primary education ≤10 years	22,470 (15.6%)	6,430 (15.5%)	5,240 (16.0%)	4,689 (15.2%)	6,111 (15.5%)
Secondary education ≤2-years	47,849 (33.1%)	13,796 (33.2%)	11,174 (34.1%)	9,801 (31.8%)	13,078 (33.3%)
Secondary education >2 years	23,991 (16.6%)	6,722 (16.2%)	5,438 (16.6%)	5,144 (16.7%)	6,687 (17.0%)
University level	50,059 (34.7%)	14,587 (35.1%)	10,871 (33.2%)	11,167 (36.3%)	13,434 (34.2%)

Abbreviations: SD indicates standard deviation; IQR indicates inter-quartile range.

^a^Gestational age specific birth weight z-scores estimated using the total study population as the reference.

**Table 2:** Associations between birth weight z-score and grip strength (Newton) in young Swedish males born at-term between 1973–1976.

	Crude		Adjusted^a^	
	Estimate (B)	95% confidence interval	Estimate (B)	95% confidence interval
**Full-cohort**				
*BW Z-score*	17.4	17.0, 17.9	17.7	17.2, 18.2
	**Crude** ^**c**^		**Adjusted** ^**b,c**^	
	Estimate (B)	95% confidence interval	Estimate (B)	95% confidence interval
**Within-families cohort**				
*BW Z-score*	13.9	10.7, 17.0	13.4	10.1, 16.6

Abbreviations: BW indicates Birth Weight.

^a^Adjusted for: parity, maternal age, maternal diabetes, maternal hypertension, cesarean section, conscription office and highest parental education.

^b^Adjusted for: same as above, excluding highest parental education.

^c^5,385 families.

**Table 3:** Linear associations between birth weight z-score and grip strength (Newton) stratified by body mass index categories at conscription in young Swedish males born at-term between 1973–1976.

	n	Crude		Adjusted^a^	
		Estimate (B)	95% confidence interval	Estimate (B)	95% confidence interval
**BW Z-score**					
Underweight (BMI <18.5)	8,980	11.4	9.6, 13.2	11.2	9.4, 13.0
Normal weight (BMI 18.5–24.9)	115,623	15.7	15.1, 16.2	15.9	15.4, 16.4
Overweight (BMI 25–29.9)	15,663	17.2	15.7, 18.8	17.4	15.8, 19.0
Obese (BMI ≥30)	3,484	18.8	15.4, 22.1	18.5	15.1, 21.9

Abbreviations: BMI indicates Body Mass Index; BW indicates Birth Weight.

^a^Adjusted for: parity, maternal age, maternal diabetes, maternal hypertension, cesarean section, conscription office and highest parental education.

**Figure 2:**
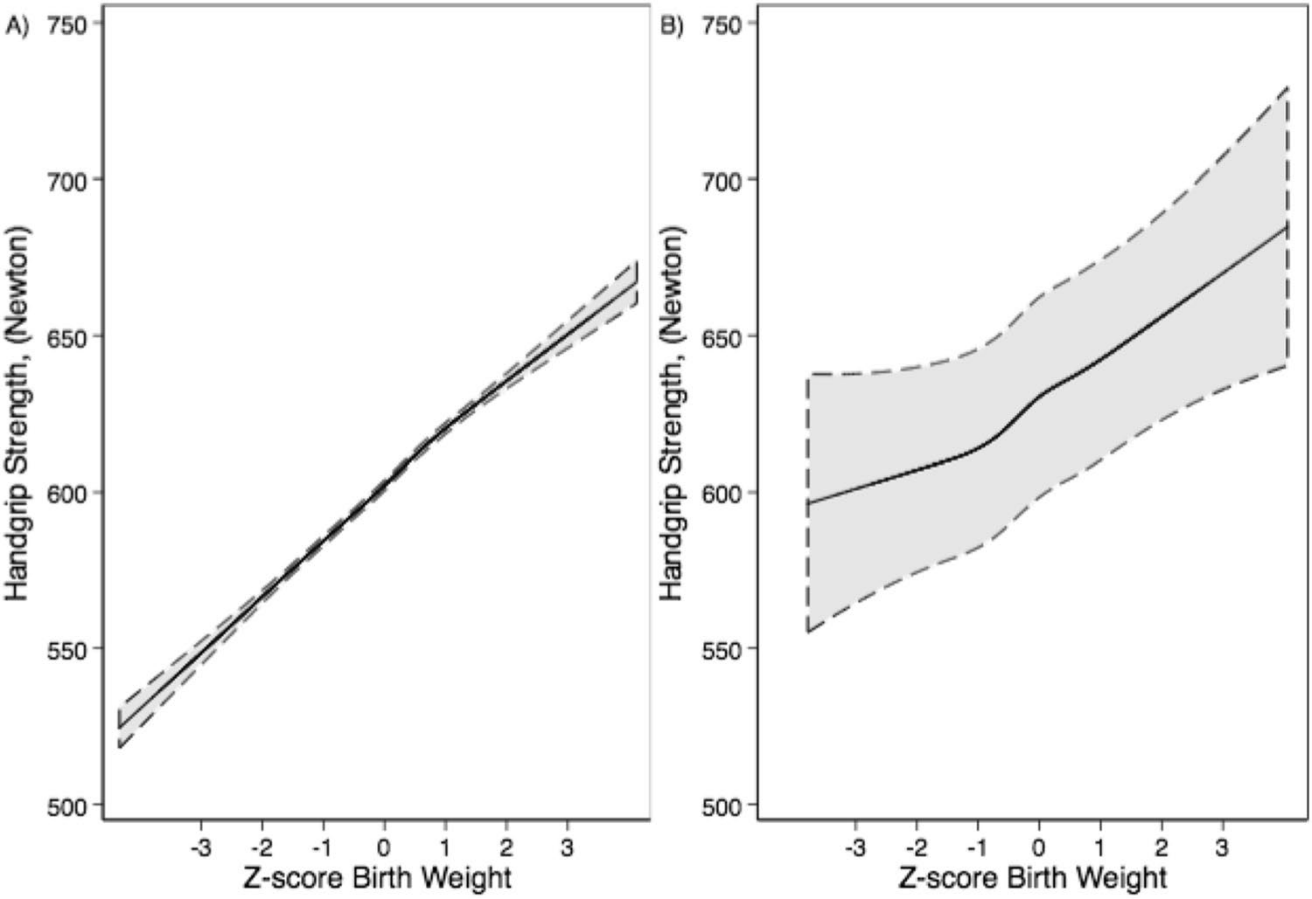
Adjusted associations, estimated with restricted cubic spline models, between birth weight z-scores and grip strength (Newton) for (**A**) the full-cohort, and (**B**) the within-families cohort (**B**) (95% confidence interval dashed).

Associations in our models are presented as crude estimates and adjusted for maternal diabetes (yes/no), maternal hypertension (yes/no), caesarean section (yes/no), parity (categorical), maternal age (continuous), highest parental education (categorical) and conscription office (categorical). We further employed restricted cubic splines with five knots at: 5, 27.5, 50, 72.5 and 95 percentiles, to assess departure from linearity (Fig. 2). All analyses were performed using Stata 14.0 (Stata Corp, College Station, TX, USA).

### Sensitivity analyses

We conducted a sensitivity analysis where we stratified the linear association between z-score birth weight, within the at-term range, and grip strength by quartiles of height in centimeter (cm) (Supplementary Table 2). The quartiles were constructed at the 25, 50 and 75 percentiles which resulted in the groups: ≤175 cm, 176–180 cm, 181–184 cm, and ≥185 cm.

### Compliance with ethical standards

The study was approved by the Regional Ethical Review Board, Stockholm (Dnr: 2016/1445-31/1). The requirement to obtain informed consent was waived by the Regional Ethical Review Board, Stockholm (Dnr: 2016/1445-31/1). All research was performed in accordance with relevant guidelines/regulations.

## Results

### Descriptive statistics

Table 1 presents descriptive data for the 144,369 individuals in the full cohort according to five categories: (i) full-cohort, (ii) high grip strength/high birth weight z-score, (iii) high grip strength/low birth weight z-score, (iv) low grip strength/high birth weight z-score and (v) low grip strength/low birth weight z-score at conscription. A similar description for the within-families cohort is presented in Supplementary Table 1. In general, most covariates such as maternal diabetes, maternal hypertension, highest parental education etc. were fairly equally distributed between the five categories, whereas birth by caesarian section was slightly elevated in the low grip strength/low birth weight z-score group.

### Birth weight, within the at-term range, and grip strength

Table 2 presents the crude and adjusted associations between birth weight z-score and grip strength (N), both for the full- and for the within-family cohort. Higher birth weight, within the at-term range, was associated with higher grip strength, with slightly weaker associations in the within-families cohort (24% reduction). In the fully adjusted model, each unit increase in birth weight z-score (1 SD) was associated with increases of 17.7 (95% CI, 17.3–18.2) and 13.4 (10.1–16.6) in N (equal to approximately 1.8 and 1.4 kilogram-force) in the full- and within-families cohort, respectively.

Figure 2 illustrates adjusted associations, estimated with restricted cubic spline models, between birth weight z-scores and grip strength (N) for both the full- and within-families cohorts. Association are in general linear in both cohorts. The detected statistical deviation from linearity (p < 0.05) can largely be explained by the large number of observations.

Table 3 presents both the crude and adjusted association between birth weight z-score, within the at-term range, and grip strength (N), stratified by four BMI categories: underweight (BMI <18.5), normal weight (BMI 18.5–24.9), overweight (BMI 25–29.9) and obese (BMI >30). Although some diminution in the underweight category, we observed consistent positive associations between birth weight z-score, within the at-term range, and grip strength across all categories of BMI. As expected, we observed no interaction between continuous BMI and z-score birth weight in the association with grip-strength (data not shown).

### Sensitivity analysis

Supplementary Table 2 presents the linear association between birth weight z-score, within the at-term range, and grip strength, stratified by quartiles of height (cm) at conscription. Although some attenuation across the whole range of height, we observed a consistent positive association between birth weight z-score, within the at-term range, and grip strength regardless of the quartile of height.

## Discussion

This total population-based study of 144,369 males showed that higher birth weight, within the at-term range (i.e. week 37 to 41), was prospectively and positively associated with grip strength in young adulthood (17–22 years) across all categories of BMI. Moreover, our within-sibling analysis, indicates that this association appears to be mainly driven by factors that are not shared between siblings. Consequently, the present study provides novel evidence to this emerging field. To the best of our knowledge, this is the first study that has examined associations of variations in the levels of grip strength, across all BMI categories, in young adulthood across different birth weights within the at-term range, using family-based analysis which provides a unique opportunity to explore whether this association is mainly driven by factors that are shared or non-shared between siblings. Although this study cannot disentangle causal pathways, grip strength may to some extent be determined already *in utero*.

Explanations for the protective and lifelong effect that birth weight, within the at-term range, seems to have on grip strength could be that the number of mammalian muscle fibers is determined at or soon after birth^22^ and influenced by nutritional status during critical periods of early development^23^. This is supported by animal^24^ and human studies^25,26^ showing a reduced muscle fiber composition in those with low birth weight. Postnatal muscle growth (e.g. via strength exercise) occurs by growth of each muscle fiber, which to some extent can compensate a deficit in fiber number^27^. However, individuals born with fewer muscle fibers will be at considerable disadvantage in terms of muscle strength later in life. In addition, low birth weight is associated with abnormal development that triggers adaptations in tissues and organs, eventually resulting in permanent muscle related physiological alterations^28^, which my result in long-lasting impairments in muscle strength^29^.

Meta-analysis data shows that grip strength has a strong genetic component with an estimated heritability of approximately 50%^30^. Likewise, a common genetic cause of both low birth weight and low grip strength cannot be excluded. However, results from our within-sibling analysis, where shared influences between siblings such as genetic factors, parental socioeconomic influences etc. are held constant, only differed marginally from analyses based on the full cohort. This provides some support to the hypothesis that shared factors between siblings most likely only explain part of the observed associations between birth weight, within the at-term range, and grip strength These findings support the notion that a poor intrauterine nutrition leading to a low birth weight for gestational age is programming grip strength later in life. However, twin-pair analyses are needed to further detangle the genetic and in utero effects that may explain the association between birth weight, within the at-term range, and later grip strength.

The observed associations between birth weight, within the at-term range, and grip strength may partly be explained by differences in upbringing between children born adequate and small for gestational age. For example, parents may choose to restrict low birth weight children’s participation in strength promoting physical activity because of perceptions of fragility^31^. Consequently, varying levels of engagement in strength promoting physical activity among the study population may to some extent explain observed association between birth weight, within the at-term range, and grip strength in young adulthood. Our finding that birth weight, within the at-term-range, is associated with grip strength in young adulthood across all BMI categories suggests that this association is not simply explained by a skeletal size effect, since larger persons tend to have larger muscles. This is further supported by our sensitivity analysis showing a consistent positive association between birth weight z-score, within the at-term range, and grip strength regardless of the quartile of height.

The study of early determinants of grip strength is of special interest since low grip strength in adolescence is strongly associated with a wide range of negative health outcomes later in life, including increased all-cause mortality rates equivalent to that for well-established risk factors such as elevated blood pressure or BMI^8^. Previous data has shown that higher grip strength essentially attenuates the negative effects from elevated BMI on mortality in men^11^. The weakest men (lowest fifth) in the most obese group has shown to have a 39% increased mortality risk (HR, 1.39; 95% CI, 1.14–1.68) and this risk was negligible among the strongest men (highest fifth) in the same most obese group (HR, 1.02; 95% CI, 0.84–1.25). Consequently, findings from this study showing that birth weight, even within at-term deliveries, is associated with grip strength in young adulthood across all categories of BMI, may be of public health interest.

The 17.7 unit increase in grip strength (N) for each SD increase in birth weight z-score reported in this study, translates into approximately a two kilogram stronger grip strength. This may have clinically important implications. For example, data from the U.K. Biobank study in over 400,000 adults aged 40–69 years has shown that every three kilogram increment in grip strength was associated with approximately 8% lower mortality^32^. Since approximately 15% of all live births are low birth weight births^33^, which are associated with lifelong health consequences^34,35^, preventing low birth weights, even within the at-term range, may have long-term effects on markers of health such as grip strength. It is of importance to state that not all low preterm or low birth weights can or should be prevented, for example spontaneously occurring births. However, recent trends towards shorter gestational lengths within the at‐term range can to some extent be attributed to the increases in the rates of planned cesarean deliveries^36^, which is a modifiable factor.

Studies investigating the associations between birth weight or gestational age and grip strength have been confined to grip strength comparisons between preterm births, not taking gestational age into account, and at-term born being used as homogenous comparator^15^. For example, a meta-analysis, including 17 studies and 21,344 individuals aged 5 to 86 years, showed that an extra kilogram of birth weight was associated with a 0.9 kilogram increase (95% C, 0.58, 1.2) in grip strength. However, none of the included studies did control for shared factors in within-sibling analyses and/or investigate the associations between birth weight, within the at-term range, and grip strength and/or whether associations varied with BMI. Thus far, only one small cohort study has demonstrated that within a twin-pair the heavier twin at birth has greater grip strength in adult life compared with the lighter sibling. In addition, the twin-pair analyses showed that associations between birth weight and adult grip strength were stronger in dizygotic than monozygotic twin-pairs, suggesting a potential influence of genetic factors, acting on both birth weight and adult grip strength^37^. Similarly, we found evidence for a stronger association between birth weight, within the at-term range, and grip strength in young adulthood in all included men compared with the within-sibling analysis including men who had one or more matchable male sibling/s.

The strengths of this study lies in the large sample size and accompanying statistical power. Furthermore, strengths include the population-based design and the long-term follow-up, where prospectively collected data at birth could be linked to grip strength at ages 17–22. We were also able to adjust for perinatal and other major determinants (e.g. birth delivery, parental education, parity etc.) of grip strength and the within-sibling analysis enabled us to disentangle whether associations were mainly driven by factors shared or non-shared between siblings. Furthermore, both exposure (birth weight within the at-term range) and outcome (grip strength) were measured using objective and standardized procedures. In fact, grip strength is the test of muscular strength that has shown the strongest evidence in relation to mortality^38–40^. Finally, we rule out confounding by gestational age and/or prematurity by using births within the at-term range.

This study is accompanied with several limitations. First, the cohort included only young adult men and the potential generalizability to women is largely unknown. Previous data has shown that the magnitude of association between birth weight and adult grip strength in women is approximately half of that seen in men^37,41^. Second, residual confounding could be present. For example, the lack of data on maternal nutrition and smoking habits (of mothers before/during pregnancy), could have had an impact on both the exposure (birth weight) and outcome (grip strength). However, smoking is more prevalent in families with low parental education. Thus, some of the effect of smoking on grip strength is accounted for in our models. Moreover, the within-families analysis accounts for habits (e.g. maternal smoking) that are consistent over time. Fourth, the within-families analysis including siblings only allow us to partly control for shared genetic factors (50%) between siblings. It is unlikely that segregating genes, potentially influencing grip strength, are randomly distributed with regards to birth weight. Fifth, since the birth factors were recorded in the 70’s, gestational age was estimated by the last menstrual period, which has limited accuracy. This may affect our precision in determining the at-term range. Sixth, register-based studies have inherited limitations, e.g. unknown data quality and possible differences in data collection between institutions. Finally, it should be noted that we did not measure engagement in strength promoting physical activity, which can influence grip strength. Hence, it is possible that the associations between birth weight, within the at-term range, and grip strength in young adulthood may be mediated by engagement in strength promoting physical activity.

## Conclusions

In summary, this large prospective cohort study has shown that higher birth weight, within the at-term range, is prospectively associated with higher grip strength in young male adults, across all categories of BMI and variations in height. Furthermore, our within-family analysis has shown that this association appears to be mainly driven by factors that are not shared between siblings, reducing the likelihood that this association is largely explained by genetics and other environmental factors shared within families. There are undeniably clinically relevant and strong prospective associations between grip strength in adolescence and later life health outcomes. Consequently, our novel findings both expand our understanding of determinants of grip strength and may have public health implications. For example, highlighting the importance of birth weight and heterogeneity among at-term deliveries, which have implications for how at-term deliveries are followed-up and monitored.

## Supplementary information

SUPPLEMENTAL MATERIAL

## Data Availability

The data that support the findings of this study are available from the Swedish National Board of Health and Welfare (Medical birth data), Swedish Defence Recruitment Agency (anthropometric and strength measures), Statistics Sweden (linking of parental data and covariate data) but restrictions apply to the availability of these data, which were used under license for the current study, and so are not publicly available. Data are however available from the authors upon reasonable request and with permission of Swedish National Board of Health and Welfare, Swedish Defence Recruitment Agency, Statistics Sweden and the Regional Ethical Review Board, Stockholm.
