# Supplementary material for: Birth weight and grip strength in young Swedish males: a longitudinal matched sibling analysis and across all body mass index ranges
Source: Sci Rep. 2019 Jul 4;9:9719. doi: 10.1038/s41598-019-46200-0 (PMC6609642; doi:10.1038/s41598-019-46200-0)
Supplement: Supplementary file 1 — SUPPLEMENTAL MATERIAL [file 41598_2019_46200_MOESM1_ESM.pdf]

SUPPLEMENTAL MATERIAL

Title

Birth weight and grip strength in young Swedish males a longitudinal matched sibling analysis and across all body mass index ranges

Authors

Viktor H. Ahlqvist B.Sc.; [1] ; Margareta Persson PhD.[2] ; Francisco B. Ortega PhD.[3, 4]; Per Tynelius PhD.[1, 5]; Cecilia Magnusson PhD.[1, 5]; Daniel Berglind* PhD.[1, 5]

[1] Department of Public Health Sciences, Karolinska Institutet, Sweden

[2] Umeå University, Department of Nursing, Sweden

[3,] PROFITH “PROmoting FITness and Health through physical activity” research group, Department of Physical Education and Sports, Faculty of Sport Sciences, University of Granada, Granada, Spain

[4]Department of Biosciences and Nutrition, Karolinska Institutet, Sweden

[5]Centre for Epidemiology and Community Medicine, Stockholm County Council, Stockholm, Sweden

Corresponding author

Daniel Berglind PhD., Department of Public Health Sciences, Karolinska Institutet, Sweden.

E-mail: daniel.berglind@ki.se

Adress: Centre for Epidemiology and Community Medicine (CES), Stockholm County's Health Care District (SLSO), Solnavägen 1E, 113 65 Stockholm

Table 1. Sample characteristics of the within-families cohort by birth weight (z-score) and grip strength (Newton).

	**Within-families cohort**	**High grip strength and High birth weight**[a]	**High grip strength and Low birth weight**[a]	**Low grip strength and High birth weight***	**Low grip strength and Low birth weight***
	N=10,791	N=3,133	N=2,533	N=2,281	N=2,844
Gestational age (weeks), mean (SD)	39.6 (1.1)	39.5 (1.1)	39.6 (1.1)	39.6 (1.1)	39.6 (1.1)
Birth weight (g), mean (SD)	3,585.2 (469.1)	3,944.6 (338.2)	3,263.9 (295.2)	3,907.1 (330.7)	3,217.4 (301.6)
Handgrip strength (N), mean (SD)	616.9 (97.3)	694.0 (70.8)	682.1 (64.1)	542.5 (50.4)	533.4 (54.3)
BMI (kg/m²) at conscription, mean (SD)	22.0 (2.8)	22.7 (2.9)	22.4 (2.8)	21.5 (2.7)	21.2 (2.7)
Height (cm) at conscription, mean (SD)	179.8 (6.4)	182.6 (6.0)	179.8 (6.0)	179.5 (6.1)	176.8 (6.0)
Age at conscription, mean (SD)	18.3 (0.4)	18.3 (0.4)	18.3 (0.4)	18.3 (0.3)	18.3 (0.3)
Maternal age at birth, mean (SD)	26.2 (4.2)	26.3 (4.2)	25.6 (4.1)	26.7 (4.3)	26.1 (4.1)
Maternal parity, median (IQR)	2.0 (1.0, 2.0)	2.0 (1.0, 2.0)	2.0 (1.0, 2.0)	2.0 (1.0, 2.0)	2.0 (1.0, 2.0)
Maternal diabetes mellitus at pregnancy, n (%)	16 (0.1%)	7 (0.2%)	1 (<1%)	7 (0.3%)	1 (<1%)
Birth by cesarean section, n (%)	559 (5.2%)	160 (5.1%)	113 (4.5%)	123 (5.4%)	163 (5.7%)
Maternal hypertension at pregnancy, n (%)	2 (<1%)	0 (0.0%)	1 (<1%)	1 (<1%)	0 (0.0%)
Parental highest level of education, n (%)					
Primary education ≤10 years	1,399 (13.0%)	376 (12.0%)	369 (14.6%)	272 (11.9%)	382 (13.4%)
Secondary education ≤2-years	3,349 (31.0%)	975 (31.1%)	840 (33.2%)	679 (29.8%)	855 (30.1%)
Secondary education >2 years	1,796 (16.6%)	544 (17.4%)	404 (15.9%)	385 (16.9%)	463 (16.3%)
University level	4,247 (39.4%)	1,238 (39.5%)	920 (36.3%)	945 (41.4%)	1,144 (40.2%)

[a]Gestational age specific birth weight z-scores estimated using the total study population as the reference

Table 2: Linear associations between birth weight z-score, within the at-term range, and grip strength (Newton), stratified by quartiles of height (cm) at conscription.

		Crude		***Adjusted***[a]	
BW Z-score	**n**	**Estimate (B)**	**CI 95%**	**Estimate (B)**	**CI 95%**
Q1 (151-175cm)	37,525	11.1	10.2 - 12.0	11.1	10.2 - 12.0
Q2 (176-180cm)	42,436	8.3	7.4 - 9.3	8.2	7.3 - 9.2
Q3 (181-184cm)	31,431	9.4	8.3 - 10.5	9.0	7.9 - 10.1
Q4 (185-208cm)	32,332	11.6	10.5 - 12.7	11.4	10.3 12.5

BW, birth weight; Q1-4, quartile 1-4.
[a]Adjusted for: parity, maternal age, maternal diabetes, maternal hypertension, cesarean section, conscription office and highest parental education.
